# Beyond community benefit: Unveiling hospitals’ comprehensive efforts to improve community health

**DOI:** 10.1093/haschl/qxaf062

**Published:** 2025-05-05

**Authors:** Cherie Conley, Aiden Swanson, Simone R Singh

**Affiliations:** Department of Systems, Population, and Leadership, University of Michigan School of Nursing, Ann Arbor, MI 48109, United States; Department of Health Policy and Management, University of Michigan School of Public Health, Ann Arbor, MI 48109, United States; Department of Health Policy and Management, University of Michigan School of Public Health, Ann Arbor, MI 48109, United States

**Keywords:** community benefit, community health, social determinants of health, nonprofit hospitals, IRS Form 990 Schedule H

## Abstract

Calls for nonprofit hospitals to clearly make their case for tax exemption are increasing. Most published research on nonprofit hospitals’ provision of community benefit relies on data reported in Internal Revenue Service (IRS) Form 990 Schedule H. This study leverages insight from hospital leaders to better understand the types of initiatives, beyond community benefit, that hospitals engage in to benefit their communities. We conducted 17 semi-structured interviews with a total of 34 hospital representatives. Three themes were identified: (1) the current IRS Form 990 Schedule H provides only limited insights into hospitals’ investments into communities, (2) health systems engage in a variety of diverse activities that benefit communities and address social determinants of health, and (3) health systems use a variety of communication channels outside of Form 990 and Schedule H to raise awareness about their contributions to community health. These findings suggest that IRS reports alone do not fully illustrate the scope of hospital initiatives to benefit communities. Internal changes in hospital practices and procedures, and external policy levers, may provide a more comprehensive picture of benefits and opportunities for improvement.

## Introduction

As vital stakeholders in their local communities, hospitals possess a wealth of resources, expertise, and influence that extends far beyond providing medical care to individual patients. Recognizing this, many hospitals have been investing in programs and activities aimed at improving the social, economic, and environmental factors that shape the health of individuals in their communities.^1^ Unlike their for-profit and government-owned counterparts, nonprofit hospitals, which represent the majority of US hospitals, are required to provide community benefits in exchange for their tax-exempt status.^2^ Their community benefit activities are subject to specific Internal Revenue Service (IRS) inclusion criteria and reporting guidelines.^3^ To report their community benefit spending, nonprofit hospitals complete IRS Form 990 Schedule H, which asks hospitals to provide detailed information on the following categories of community benefit: financial assistance and means-tested government programs, community health improvement, health professions education, subsidized health services, research, cash and in-kind contributions, community-building activities, as well as bad debt, Medicare, and collection practices.^4^

Prior research studies as well as policy groups and government officials have questioned whether nonprofit hospitals are doing enough to address the social drivers of patient and community health.^5^ Currently, there is no federal mandate for how much nonprofit hospitals have to spend on community benefit overall or in specific community benefit categories in return for tax exemption, nor are hospitals required to report the value of their tax exemption.^6,7^ In fact, of the total community benefit expenditures, the majority—an estimated 85%—is allocated to clinical and patient care services only (means-tested government programs, bad debt, Medicare, subsidized health services).^8^ The other 15% is spread out over all other programs—community health improvement, health professions education, research, cash and in-kind contributions, and community-building activities. An estimated 4% of community benefit spending and less than 1% of total hospital expenditures are allocated to community health improvement.^2^

While there is no current federal mandate for full disclosure, and tax-exempt status has rarely been revoked, the calls for nonprofit hospitals to clearly make their case for tax exemption are increasing.^8^ At the federal and state level, legislators have requested investigations into some hospitals’ community benefit practices, applied more stringent assessment of community benefit programs’ alignment with the goals of improved community health,^9^ increased oversight by mandating a minimum threshold for community benefit spending, and, as was the case recently with several hospitals in Pennsylvania, have supported revocation of tax exemption^10,11^ when hospitals were deemed as not having adequately provided benefit to the community.

Most published research on nonprofit hospitals’ provision of community benefit relies on data reported in IRS Form 990 Schedule H. Schedule H contains key information about nonprofit hospitals’ community benefit investments in their communities^12^ and must adhere to specific guidelines set by the IRS.^13^ For instance, if community benefit programs benefit hospital employees, even if they are members of the community, it may not be counted as community benefit.^14^ Also, some initiatives that address what may be considered root causes of poor health, such as environmental conditions, transportation, or workforce development, must be reported separately and receive special approval to be credited as community benefit by the IRS.^2,15^ Given the very specific reporting requirements, public-facing IRS Form 990 Schedule H may not provide a full picture of hospitals’ efforts to improve community health.

To better understand the types of initiatives that nonprofit hospitals engage in to improve the health of their communities, this study leverages insight from hospital and health system leaders to (1) assess the usefulness of IRS Form 990 Schedule H for the purpose of community benefit reporting, (2) provide insights into hospitals’ community contributions beyond the investments reported on IRS Form 990 Schedule H, and (3) explore communication channels used by hospitals in addition to IRS Form 990 to communicate the types and impact of their community contributions.

Data from this study will be used to provide more nuanced insight into hospitals’ work benefiting communities beyond what may be entailed with IRS 990 reporting requirements. We also expect this work to inform federal, state, and institutional practice and policy regarding feasible strategies to assess hospitals’ efforts to address social determinants and improve community health.

## Data and methods

### Data and sample

We partnered with The Academy Advisors (TAA) at the Health Management Academy—a national organization of health care leaders created to address critical issues in the health care industry—to conduct key informant interviews with leaders in hospital community benefit. Our inclusion criteria were TAA member hospitals or health systems that have engaged in initiatives designed to address social needs and social determinants of health work for at least 3 years, and any administrators, executives, or leaders familiar with their organization's community benefits, social needs, and upstream social determinants of health initiatives. Invitations to participate in the study were sent via email by the study team to systems identified by TAA as meeting all inclusion criteria.

Interviews were conducted via Zoom between December 2023 and April 2024. The interview guide (see Appendix A [to access the Appendix, click on the Details tab of the article online]) included questions about the activities that the representative organizations engaged in aimed at addressing social determinants of health—whether or not they were counted and reported as community benefit on the organization's IRS Form 990 Schedule H, and how they engaged with stakeholders to communicate their investment and commitment to addressing community health. We also asked interviewees to identify specific measurable indicators that reflect their investment in initiatives that benefit the communities they serve.

### Analysis

Interviews were recorded, then transcribed and analyzed, by the research team using Microsoft Office Word and Excel. Conventional content analysis was used to analyze the transcripts. Codes were developed within 2 a priori interview categories based on the following study aims: (1) explore hospital contributions to community health beyond the IRS Form 990 and (2) explore how hospitals communicate their contributions to community health. A second study team member reviewed 30% of transcripts. Any differences in code identification were discussed and resolved by the 2 study team members to establish a final set of codes, which were then grouped to form themes and subthemes across all of the transcripts to address study aims.

### Limitations

This study has several limitations. First, the sample for this study was limited to mostly large nonprofit hospitals and health systems in primarily urban areas. Thus, study findings might not reflect the experience of smaller hospitals as well as hospitals located in rural areas. Another limitation is that we only worked with hospitals who were TAA member organizations. TAA members may share common perspectives and experiences related to community benefit that are not reflective of other nonmember health systems, which would also limit the generalizability of our results.

## Results

We conducted 17 semi-structured interviews with a total of 34 hospital leaders, community benefit managers, and administrators from 14 TAA member health systems. Interviewees represented all regions of the United States and a variety of departments within health systems, including population health, government relations, finance, community benefit, clinical, and leadership (see Table 1).

**Table 1.** Description of sample hospitals and interviewees.

Health systems (*n* = 14)	Interviews (*n* = 17)	Interviewees (*n* = 34)	Region	Hospital size (no. of beds)	Departments represented
#1	2	5	Northeast	1000–3000	Community benefit, government affairs, health equity
#2	1	2	West	>3000	Public relations, community benefit, community/population health
#3	1	3	Northeast	>3000	Public relations, community/population health, finance, community benefit, operations
#4	2	2	West	<1000	Health equity, government affairs
#5	1	1	West	>3000	Public relations, community affairs
#6	1	3	South	<1000	Community affairs, government relations, public policy, finance
#7	1	3	East	1000–3000	Government affairs, community affairs, health equity
#8	2	5	South	>3000	Public policy, community/population health
#9	1	2	Northeast	<1000	Quality, government relations, community health
#10	1	1	Midwest	<1000	Government relations
#11	1	3	Midwest	<1000	Community affairs, health equity
#12	1	2	Midwest	1000–3000	Government affairs, community affairs
#13	1	1	East	1000–3000	Community health, community benefit
#14	1	3	Midwest	>3000	Health equity, analytics, community health

Source: Authors’ analysis of data from semi-structured interviews.

Three themes were identified: (1) the current IRS Form 990 Schedule H provides only limited insights into hospitals’ investments into communities, (2) health systems engage in a variety of diverse activities that benefit communities and address social determinants of health, and (3) health systems use a variety of communication channels outside of Form 990 and Schedule H to raise awareness about their contributions to community health.

### Theme 1: Current IRS Form 990 Schedule H provides only limited insights into hospitals’ investments into community health

All interviewees except for 1 stated that they did not or could not count all of their activities and initiatives aimed at improving community health on IRS 990 Form Schedule H, even though the initiatives required significant hospital resources and, ultimately, they believed, provided great benefit for the communities they serve. Therefore, many interviewees stated that IRS Form 990 was not a true depiction of what they were actually investing to benefit the community.

Community health–focused initiatives were not reported on IRS Form 990 for 3 main reasons: (1) challenges related to tracking some of their investments in work to benefit communities, (2) challenges related to systematically capturing and reporting work that was being done, and (3) challenges related to limitations of IRS Form 990 reporting structure and community benefit inclusion requirements (Table 2).

**Table 2.** Theme 1 and subthemes.

	Representative quotes
Theme 1: Current IRS Form 990 Schedule H provides only limited insights into hospitals’ investments into community health	“…when you have an organization…that is dedicating time to putting coalitions together to advocate for key policies or just convenings to try to understand where do we have gaps in the services that are provided in our communities…. And also the public policy leadership…we kind of sometimes set the tone or set the pace for different policies. And so that's not necessarily captured in our community benefit work.”—Interviewee #10
Subthemes	
1. Challenges related to accurately measuring investments	“The other thing that's not captured in our 990 is the amount of time that we spent building relationships with people…just talking to people, getting to know them….So that's the kind of trust building activities…. And it is foundational to the work. We can't do the work if we don't spend the time. So that's not counted in the 990.”—Interviewee #14 “All of that great wonderful equity work we're doing that has some real impact on probably like our highest area of need patients. You are not pulling that out of the 990. And honestly like I'm not putting in an expense for that whole team that meets routinely that figures out those dashboards, that figures out those interventions.”—Interviewee #1
2. Challenges related to capturing and reporting investments	“I mean, inherent in a huge system, there's not a lot of coordination across different divisions, I would say. And even within our hospital, I mean, outside of our schools, I have an emergency department team that provided the medical services at [a large citywide event] this year. And that's not going to appear in a community benefit report because although I've told someone about it, it's just there's not a super coordinated effort across our divisions to report out all of these different impacts.”—Interviewee #4
3. Limitations of IRS Form 990 and Schedule H structure and community benefit inclusion criteria	“We're not trying to fluff anything up. I think that what we find is that sometimes we under report all the things that we're doing because, oh, that won't count. And it's like, ‘No, that absolutely matters.’ And even if it doesn't go to a dollar amount, it goes to our story, which is equally if not more valuable because what people misunderstand is the health system's commitment to community and the ways in which we show up.”—Interviewee #11

Source: Authors’ analysis of data from semi-structured interviews.

Abbreviation: IRS, Internal Revenue Service.

#### Subtheme 1: Challenges related to accurately tracking investments in the work to benefit communities

The investment that interviewees found most difficult to measure was human capital. Human capital includes resources needed to coordinate programs across divisions within the health system and hours spent building coalitions and partnerships—policy, legal, and community. Participants emphasized that to do their work in ways that will embed equity in the organizations’ operations and processes and also be meaningful to their community, it takes resources and time and there is no shortcut. However, consistently tracking these hours as well as the time and resources invested into planning or designing community initiatives is sometimes lacking.

#### Subtheme 2: Challenges related to reporting investments into social determinants of health initiatives

Interviewees stated that their organizations had not implemented systematic processes to ensure that all departments report their activities to aggregate them for federal or state reporting purposes. Therefore, even if work is done, it is not always being captured and reported to a central hub to be included on IRS Form 990.

In response to this issue, interviewees discussed various approaches to create more coordinated, centralized, and standardized methods for reporting community benefit activities within their systems. This often was done via new dashboards that included user-friendly data-entry portals or online reporting sheets and shared documents that employees could use to enter their work or hours. In conjunction, many new systems required administrators to spend time educating employees on how to consistently capture and report everything that they do to benefit communities.

#### Subtheme 3: Challenges related to limitations of IRS Form 990 Schedule H and community benefit inclusion criteria

Interviewees discussed the challenges with both the community benefit inclusion criteria and the structure of IRS Form 990. Interviewees often felt like they could not adequately describe all of the resources and coordination invested to implement and sustain programs that benefit communities and address social determinants of health on IRS forms.

Interviewees also discussed the impact of specific IRS community benefit credit stipulations, which prevented some of their investments from legally being credited as a community benefit. For instance, 1 interviewee described how their hospital financed the construction of a new high school, and multiple interviewees described partnerships with community colleges and high schools, establishment of new scholarship programs, writing grants, and targeted recruitment of individuals from less-resourced communities for these educational and employment opportunities. Although the communities benefit, because the hospital may also benefit if the program participants become hospital employees, it cannot be counted by the IRS as community benefit.

In response, interviewees described being very conservative with what they report to ensure they followed IRS guidelines, even if that meant leaving out some of their programs.

### Theme 2: Health systems engage in a variety of diverse activities that benefit communities and address social determinants of health

Interviewees mentioned a variety of initiatives aimed at addressing upstream social determinants of health as well as more downstream social needs. The main areas of focus were (1) workforce and economic development; (2) supplier diversity; (3) community-building activities focused on food insecurity, housing, and transportation; and (4) health care access. Interviewees stated that, while not all of the initiatives could be reported as community benefit—for instance, those that benefited hospital employees, those that received grant funding, and those created to increase employment at the hospital—they all were considered beneficial to the community (Table 3).

**Table 3.** Theme 2 and subthemes.

	Representative quotes
Theme 2: Health systems engage in a variety of diverse activities that benefit communities and address social determinants of health	“Workforce development is one that we've leaned really heavily on for two reasons. Financial stability shows up in almost all of our community health needs assessment across our footprint. And two, we have a workforce crisis in terms of shortage…. We're 60% rural.…Not only does it raise the tide of economics within the community, but… if they already have ties to the community, know their community, love their rural community, have family there, it's a little different than recruiting and bringing people in.”—Interviewee #5
Subthemes	
1. Workforce and economic development	“…those multimillion dollar investments aren't counted in community benefit because there's a tie of either these are going to [our] employees or folks that go through it and make a commitment to work for us for a certain number of years. We do have education, academic and workforce investments that do count to some extent, but there are some significant restrictions on what counts in the community benefit and what doesn't.”—Interviewee #6
2. Supplier diversity	“… [I was] visiting with a friend who was diagnosed with lung cancer…. So, I came back and I said, do we have hair care products for black women in our hospitals? And the answer was, no…I mean, I know it's a little thing, but it makes a difference in everyday life for people. And so, now we have our procurement people specifically looking locally, if there's someone we could purchase haircare products from. So, you don't really count that in your community benefit, but it's something that's very important in our communities.…”—Interviewee #7
3. Community-building initiatives	“You can't deal with someone's diabetes when they don't have secure housing, secure source of food. It's like, no, we gotta meet them where they're at right now. They care about their dog and their shopping cart and where they're gonna go. Right?…. And so it's that kind of work that I think hospitals need to do more of.”—Interviewee #2
4. Health care access	“…we work with local shelters, and it allows us to bring our expertise, our medical clinical care, our home care into the shelter. Oftentimes shelters require individuals to leave for the day. [Our] patients… are allowed to stay at the shelter outside of the normal rules… we've seen a 70% reduction in ED [emergency department] utilization, a reduction in total cost of care. And more importantly, our patients report a better quality of life.”—Interviewee #9

Source: Authors’ analysis of data from semi-structured interviews.

#### Subtheme 1: Workforce and economic development

Multiple systems described extensive efforts to develop training and educational programs to improve employment opportunities in the communities they served, often in response to community-expressed needs for economic stability. These investments included building new schools in economically depressed neighborhoods, providing college scholarships, and providing intensive support for less-advantaged students by addressing social needs such as lack of transportation, food, or school supplies like laptops. However, most of their efforts in workforce and economic development are not automatically counted as community benefit according to IRS requirements.

#### Subtheme 2: Supplier diversity

Several of the interviewees discussed their systems’ efforts to increase supplier diversity by hiring locally and from women-, veteran-, and minority-owned businesses. One system discussed building a new hospital where a blighted and abandoned strip mall was. Not only did it provide a new face of the community, but during its construction, they hired local companies and later employed people from the area to run the facility. Another system hosts conferences annually in their hospital for minority- and women-owned local businesses and provides grant investments to those businesses. Similar to workforce and economic development, however, all actions that they felt benefited the community could not be counted in their community benefit expenses.

#### Subtheme 3: Community-building initiatives focused on food security, housing, and transportation as social determinants of health

Several of the examples provided focused on youth. One health system began a school clinic that also had a food pantry. Students were allowed to go shopping in the food pantry and take groceries home. Another interviewee described a program that helps youth in the community facing criminal charges that lead to a suspended driver's license. In response to their community saying that youth and employment was a major issue, they started a program to walk young people through the process of getting their license reinstated for work and held discussions with the community on how to avoid similar situations in the future.

#### Subtheme 4: Health care access

Interviewees also reported that building new health care facilities, clinics, and mobile clinics in low-resourced areas like rural communities, or for underserved populations like the LGBTQ+ community, was a reflection of their commitment to equity and caring for the underserved.

### Theme 3: Hospitals use a variety of approaches outside of IRS Form 990 Schedule H to communicate their contributions to community health

Interviewees agreed that it was crucially important to raise awareness about their commitment to community health and well-being. Most interviewees stated that IRS Form 990 was not a useful tool to communicate their community benefit efforts, in part because most of their stakeholders were not regular consumers of Form 990 and may not even know where to find it. In addition, they stated that often only the financial contributions are used to judge hospitals’ contributions while the information provided in the narrative is missed (Table 4).

**Table 4.** Theme 3.

	Representative quote
Theme 3: Hospitals use a variety of approaches outside of IRS Form 990 and Schedule H to communicate their contributions to community health	“There's something you lose when you put a static document on a website, right? So it's being in community, it's participating in round tables and actually talking about our work. It's, so yes, I think there's more that could be done, but again, it's, I think it's more that face-to-face communication and actually talking and hearing from community about what the needs are and then and talking about ways we show up and engage.”—Interviewee #2

Source: Authors’ analysis of data from semi-structured interviews.

Abbreviation: IRS, Internal Revenue Service.

Additional communication channels used by study participants included state-required community benefit reports, fact sheets and infographics, local news outlets and events, meetings with legislators, community events, hospital websites, social media, and word of mouth. Communication strategies that included personal stories of people affected by their programs were effective. Getting into the community and directly speaking with people was considered essential. Multiple interviewees stated that they are in the process of learning how to become more effective using social media platforms to share health-related information.

## Discussion

Interviews with 34 hospital leaders and key informants from 14 US nonprofit hospitals and health systems provided insight into the initiatives their organizations invested in to improve community health. The hospitals and health systems interviewed for this study engage in a wide variety of initiatives to improve community health that are not always reported on IRS Form 990 Schedule H due to multiple challenges related to tracking and reporting investments and community benefit inclusion criteria. In fact, to ensure they maintain compliance, some systems err on the side of more conservative reporting of initiatives that they may see as beneficial to the community but may not fall clearly within the guidelines for inclusion as community benefit. Given these challenges, IRS Form 990 Schedule H is limited in its ability to provide a comprehensive picture of nonprofit hospitals’ community benefits. Both internal changes in hospital practices and procedures, and external policy levers, may help alleviate some of these challenges.

Internally, hospitals can develop (1) internal reporting systems that allow for systematic capture of all activities and initiatives that are reportable on IRS Form 990 Schedule H; (2) consistent, layered, and innovative communication strategies to highlight activities and initiatives that benefit the community, irrespective of whether the activities or initiatives are reportable on IRS Form 990 Schedule H; and (3) peer learning opportunities to share and refine IRS Form 990 reporting best practices.

Previous studies have indicated that changing IRS reporting guidelines and inclusion requirements for community benefit might increase hospitals’ investments in community-building initiatives.^15^ Our study showed that, while hospitals did not support additional IRS reporting requirements, expanding IRS guidelines for what is included as community benefit may address the frustrations that hospital leaders report with respect to not being able to count as community benefit some of the work they do that they see as having great potential to affect some of the root causes of poor health. For instance, currently, hospitals have to provide separate and additional justification for why some social determinants–focused community-building activities should be counted as community benefit. This could be a disincentive to report or even engage in these activities. One suggestion is for IRS guidelines to explicitly state that community-building activities focused on social determinants of health do fall within community benefit requirements and may be reported as such.^2,16^ In addition, this may provide additional opportunities for hospitals to share the breadth of their work, or potentially incentivize hospitals to expand efforts to improve community health.

Hospital leaders know who their key stakeholders are and have developed strategies and processes to increase awareness of their commitment to community health through multiple communication channels outside of IRS Form 990 Schedule H. In the future, however, given the current concern over community investments, the efficiency and accountability of the health care sector in general, and the use of new technology such as artificial intelligence (AI) to scour large sets of data, public interest in and scrutiny of nonprofit hospitals may continue to grow. Therefore, hospitals may, in fact, benefit from new state or federal policy that encourages adoption of easily accessible, publicly facing annual impact reports. The report could be used alone or to supplement information reported to the IRS to give a more nuanced and comprehensive understanding of how hospitals invest in and impact their communities, as well as highlight opportunities for growth.

Finally, hospital and health system representatives stated that the composition of community benefit spending might be a better measure of their commitment to community health and overall impact than the total amounts spent on community benefit. For instance, similar to the example provided by 1 interviewee, a hospital located in a community with few or low-wage jobs and less-resourced schools may develop a partnership with a high school and community college, and provide funding for transportation, tutoring, and food for students enrolled in their program. The program helps students improve test scores, graduate, and matriculate through community college or another training program to obtain a health-related degree. This ultimately leads to better employment opportunities, wages, and overall health for the individual and community if scaled up to include multiple organizations.

However, one of the most popular strategies to assess how committed hospitals are to addressing social care and community health is to examine and compare quantitatively hospitals’ investments using IRS tax forms. In the example above, if the hospital guarantees a job at their institution and requires students to work for them after graduating, it would not count as community benefit because the hospital ultimately is benefiting. Therefore, the investments may not be captured quantitatively in IRS reporting for community benefit, even though it may have a positive impact on community well-being and health.

While hospital representatives did not discuss any baseline threshold for community benefit investment, 1 interviewee noted that every single health system in their state had been able to meet or exceed their state-mandated minimum. However, research has also shown decreases in community benefit spending for some hospitals after a state mandate.^16^ Therefore, further studies on the effects of various approaches to state community benefit spending minimums on hospitals’ willingness and ability to invest in and impact community health are thus needed.

## Conclusion

The national shift towards value-based care, new guidelines by the Centers for Medicare and Medicaid Services and the Joint Commission on Accreditation of Healthcare Organizations on social needs screening, and increased state and federal scrutiny of nonprofit hospitals’ tax exemption are significant developments in the health care sector. They are significant factors driving community health investment by nonprofit hospitals and health systems.

Yet, hospitals and health systems are grappling with how to assess and communicate all of the activities and initiatives they invest in to address social factors that shape health and community well-being.^17^ This study provides insights, from the perspective of hospital and health system leaders, into the variety of ways that their organizations are addressing community health beyond community benefit, the strategies they use to communicate their efforts beyond the federally required IRS Form 990 Schedule H, and policy and procedural opportunities for improvement.

## Supplementary Material

qxaf062_Supplementary_Data

## Data Availability

The data that support this study are not publicly available due to privacy or ethical restrictions.
