# Supplementary material for: Beyond community benefit: Unveiling hospitals’ comprehensive efforts to improve community health
Source: Health Aff Sch. 2025 May 5;3(5):qxaf062. doi: 10.1093/haschl/qxaf062 (PMC12050685; doi:10.1093/haschl/qxaf062)
Supplement: qxaf062_Supplementary_Data [file qxaf062_supplementary_data.zip › TAA_Appendix.Supplemental Material_HealthAffairsScholar.docx]

**Appendix A**

**Aim 1:** **Interview questions**

The goal of our interview is to develop a set of key indicators of hospitals’ community impact that expand beyond the community benefit information reported on hospitals’ IRS Form 990.

**Demographic questions:**

- What is your current position/role?
- How long have you been with the organization?

**Investments in the SDOH:**

- We are specifically interested in hospitals’ contributions to upstream SDOH, such as housing, employment, education, and neighborhoods. Could you share a few of the most robust or long standing examples of SDOH-related activities, that your organization engages in?
- For the activity you just described, can you identify a specific measurable indicator that could be obtained for a hospital from either public or non-public sources to measure/report the activity?
- Does your organization currently publish information on this specific measure/indicator? If so, where? If yes, why? If not, why not?
- For the activities you described, are they all captured on Form 990 and counted as community benefit?
- For those activities not included, why are they unable to be captured on the 990 and counted as community benefit?

**Reporting community investments on IRS Form 990 and beyond:**

- Who are the stakeholders you want to know about your initiatives?
  - Do you think the stakeholders you want to know about your activities, actually do know about the extent of your activities and impact?
- Does Form 990 allow you to provide a comprehensive picture of your organization’s community benefit/impact?
- To provide a more comprehensive picture of your organization’s community benefit/impact, what modifications would need to be made to Form 990? (Individual hospital vs. group reporting)
- What other reporting format/method is most effective for communicating to stakeholders the impact you have had in communities?
